# Human cord blood progenitors with high aldehyde dehydrogenase activity improve vascular density in a model of acute myocardial infarction

**DOI:** 10.1186/1479-5876-8-24

**Published:** 2010-03-09

**Authors:** Claus S Sondergaard, David A Hess, Dustin J Maxwell, Carla Weinheimer, Ivana Rosová, Michael H Creer, David Piwnica-Worms, Attila Kovacs, Lene Pedersen, Jan A Nolta

**Affiliations:** 1 Department of Molecular Biology, Department of Hematology and Institute of Clinical Medicine, Aarhus University, Aarhus, Denmark; 2 Program in Regenerative Medicine, Krembil Centre for Stem Cell Biology, Vascular Biology Group, Robarts Research Institute and the University of Western Ontario, London, ON, Canada; 3 Department of Molecular Biology and Pharmacology, Molecular Imaging Center, Mallinckrodt Institute of Radiology, Washington University School of Medicine, St Louis, MO, USA; 4 Department of Surgery, Center for Cardiovascular Research, Washington University School of Medicine, St Louis, MO, USA; 5 Division of Oncology, Hematopoietic Development and Malignancy Program, Washington University School of Medicine, St Louis, MO, USA; 6 Department of Pathology, Umbilical Cord Blood Bank, Cardinal Glennon Children's Hospital, St Louis, MO, USA; 7 Department of Internal Medicine, Stem Cell Program and Institute for Regenerative Cures, University of California, Davis, Sacramento CA, USA

## Abstract

Human stem cells from adult sources have been shown to contribute to the regeneration of muscle, liver, heart, and vasculature. The mechanisms by which this is accomplished are, however, still not well understood. We tested the engraftment and regenerative potential of human umbilical cord blood-derived ALDH^hi^Lin^-^, and ALDH^lo^Lin^- ^cells following transplantation to NOD/SCID or NOD/SCID β2m null mice with experimentally induced acute myocardial infarction. We used combined nanoparticle labeling and whole organ fluorescent imaging to detect human cells in multiple organs 48 hours post transplantation. Engraftment and regenerative effects of cell treatment were assessed four weeks post transplantation. We found that ALDH^hi^Lin^- ^stem cells specifically located to the site of injury 48 hours post transplantation and engrafted the infarcted heart at higher frequencies than ALDH^lo^Lin^- ^committed progenitor cells four weeks post transplantation. We found no donor derived cardiomyocytes and few endothelial cells of donor origin. Cell treatment was not associated with any detectable functional improvement at the four week endpoint. There was, however, a significant increase in vascular density in the central infarct zone of ALDH^hi^Lin^- ^cell-treated mice, as compared to PBS and ALDH^lo^Lin^- ^cell-treated mice.

**Conclusions:**

Our data indicate that adult human stem cells do not become a significant part of the regenerating tissue, but rapidly home to and persist only temporarily at the site of hypoxic injury to exert trophic effects on tissue repair thereby enhancing vascular recovery.

## Introduction

Acute myocardial infarction (AMI) and the resulting complications are a leading cause of morbidity and mortality in the Western world. While conventional treatment strategies for AMI may efficiently alleviate symptoms and hinder disease progression, recovery of lost cells and tissue is rarely achievable. Transplantation of primitive progenitor cells of hematopoietic, mesenchymal, and endothelial lineages have, however, been found to enhance endogenous tissue repair in small animal disease models and to improve overall function of the affected tissues in early phase clinical trials[1]. The exact mechanism of repair is not known but may involve paracrine signaling by the donor cells or direct replacement of damaged tissue by donor cells[2].

Stem and progenitor cells derived from hematopoietic tissue have attracted much attention as a source of transplantable cells for cell-based regenerative therapy. Hematopoietic, mesenchymal, and endothelial progenitors have been identified in human bone marrow (BM) and umbilical cord blood (UCB) [3-5]. All three progenitor populations can be simultaneously isolated from human BM based on the expression of the cytosolic enzyme aldehyde dehydrogenase (ALDH)[6], although the relative contributions of the different sub-populations and consequently their relative therapeutic contribution may vary between the different cell sources. We and others have found that lineage depleted (Lin^-^) cells from BM and UCB that express high levels of ALDH (ALDH^hi^Lin) have superior long term repopulating potential in the hematopoietic tissues of NOD/LtSz-scid/scid (NOD/SCID) mice whereas lineage depleted cells that express low levels of ALDH (ALDH^lo^Lin^-^) are virtually devoid of long term repopulating potential in spite of an apparent overlap in expression of the putative human hematopoietic stem cell marker CD34 between the two populations [7-10]. Furthermore, as few as 2 × 10^5 ^ALDH^hi^Lin^- ^cells purified from UCB can engraft multiple tissues in the β-glucuronidase (GUSB) deficient NOD/SCID/MPSVII mouse model, including the pancreas, retina, lung, liver, kidney and heart at 10-12 weeks post transplantation[11].

Xenotransplantation of human hematopoietic stem cells and progenitor cells to immune deficient mice is extensively used to study human hematopoiesis and diseases involving the hematopoietic system[12]. The studies of diseases of solid organs using xenotransplantation models is, however, hampered by the lack of simple and sensitive methods for identifying human donor cells, an issue which we addressed in the current studies. We adapted the left anterior descending (LAD) coronary artery occlusion model of AMI recently described by van Laake et al[13] to highly immune deficient NOD/SCID and NOD/SCID β2-microglobulin null mice (NOD/SCID β2m null). The NOD/SCID β2m null mouse strain is deficient in the expression of the MHC class I associated cell surface protein β2-microglubulin (β2m), which is normally expressed on all nucleated cells[14]. Engrafting donor cells can thus easily be detected by immune staining for β2m.

Macroscopic evaluation of donor cell distribution to various organs following global or localized delivery is key to understanding the dynamics of stem cell engraftment in target tissues and has been described using labeling with radionuclides, fluorescent dyes, or bioluminescent or fluorescent reporter proteins[15,16]. We have recently documented that engrafting human donor cells can be visualized in situ without adversely affecting cell viability and engraftment potential by a combination of nanoparticle labeling and whole organ fluorescent imaging[17]. Using a similar approach, we have in the present study: 1) evaluated donor cell distribution to multiple organs, including the infarcted heart, at 48-72 hours post transplantation and 2) analyzed long term engraftment in multiple organs and the infarct zone as well as the regenerative effects of cell treatment by molecular and mechanistic approaches at four weeks post transplantation. By the combined nanoparticle labeling and whole organ fluorescent imaging, we found a more pronounced infarct-specific distribution of ALDH^hi^Lin^- ^stem cells, as compared to committed progenitor cells at 48-72 hours post transplantation. At four weeks post transplantation, ALDH^hi^Lin^- ^cells engrafted multiple organs, including the heart, liver and kidney, at higher frequencies than ALDH^lo^Lin^- ^cells. Under these highly permissive conditions for human cell engraftment, we found no donor derived cardiomyocytes and only few endothelial cells of donor origin at four weeks. Cell treatment was not associated with a significant improvement in cardiac performance at four weeks. There was, however, a significant increase in the vascular density of large caliber vessels in the central infarct zone of ALDH^hi^Lin^- ^cell-treated mice, as compared to PBS and ALDH^lo^Lin^- ^cell-treated animals.

## Materials and methods

### Mice

NOD/SCID and NOD/SCID β2m null mice (originally from Jackson Laboratories, Bar Harbor, ME) were bred and maintained at the animal facilities at the Washington University School of Medicine. All animal experiments and protocols were approved by the animal studies committee at Washington University School of Medicine, and conducted in compliance with the *Guide for the Care and Use of Laboratory Animals *published by the US National Institutes of Health (NIH Publication No. 85-23, revised 1996), and all University requirements.

### Human cell purification

Umbilical Cord Blood (UCB) that failed to meet the minimal total nucleated cell count was obtained from the cord blood banking facility at Cardinal Glennon Children's Hospital, St Louis, MO, and used in accordance with the ethical guidelines at Washington University School of Medicine and the principles outlined in the Declaration of Helsinki. Mononuclear cells (MNCs) were isolated from UCB by Hypaque-Ficoll centrifugation (Pharmacia Biotech, Uppsala, Sweden). MNCs from different cord blood samples were pooled (24 cords were used in total) and lineage depleted or enriched for CD34^+ ^cells as previously described[8]. Briefly, UCB MNCs were incubated with a human-specific lineage depletion antibody cocktail or anti human CD34 antibody followed by magnetic bead labeling before negative or positive selection, respectively, on an immunomagnetic separation column, according to the manufacturer's directions (Stem Cell Technologies, Vancouver, BC, Canada).

### FACS sorting of aldehyde dehydrogenase high and low expressing cells

Cells to be sorted were cultured overnight in X-Vivo 15 media (Lonza Group, Basel, Switzerland) on RetroNectin coated plates (25 μg/cm^2^; Takara Bio INC., Otsu, Japan) in the presence of recombinant human SCF, Flt3-L and TPO (all 10 ng/ml, R&D Systems, Minneapolis, MN) and nano-particles in selected experiments as indicated below. Total cells were detached on the following day by gentle washing with Cell Dissociation Buffer (CDB, Invitrogen, Carlsbad, CA) and purified according to their levels of ALDH activity by staining with the Aldefluor reagent (Aldagen, Durham, NC), according to the manufacturer's specifications. Briefly, Aldefluor substrate (0.625 μg/mL) was added to 1 to 5 × 10^6 ^Lin^- ^cells/mL suspended in Aldefluor assay buffer and incubated for 20 to 30 minutes at 37°C. Cells were then FACS sorted on a MoFlo (BD, San Jose, CA) according to high and low Aldefluor signal as described [8].

### Whole organ fluorescent imaging

#### 655 nm fluorescent emitting nano-particle labeling

Human UCB Lin^- ^or CD34^+ ^cells were incubated with 655 nm fluorescent Quantum Dot nano crystals (QD655, Invitrogen) in cell media (X-Vivo with recombinant human SCF, Flt3-L and TPO (all 10 ng/ml)) in the presence of 0.1 nM protamine sulphate for 15 min followed by overnight incubation in cell media at 10^6 ^cells/well on Retronectin coated non-tissue culture treated 24 well plates at 37°C and 5% CO_2_. The following day the Lin^- ^cells were then detached by gentle washing with CDB and resuspended in PBS and sorted according to high or low expression of ALDH as described above. The cells were then subjected to a second round of labeling overnight as described. CD34+ sorted cells were labeled in parallel but without sorting for ALDH activity.

#### 750 nm fluorescent emitting nano-particle labeling

The 750 nm fluorescently labeled paramagnetic Feridex iron nanoparticle protocol was essentially identical to the 655 nm nano-particle labeling protocol with the following modifications: Human UCB Lin^- ^cells were only subjected to a single round of labeling followed by sorting for high and low expression of ALDH as described. Labeled and sorted cells were incubated overnight in cell media without further labeling.

#### Transplantation of nano-labeled cells

Cells to be transplanted were detached on the following day by gentle washing with CDB and maintained in cell media until transplantation. NOD/SCID or NOD/SCID β2m null mice to be transplanted were subjected to AMI on the day before transplantation as described[18] and transplanted with QD655 or Feridex750 labeled cells (2 × 10^6 ^CD34^+^, 1.6 - 4 × 10^5 ^ALDH^lo^Lin^-^; 2.3 - 4 × 10^5 ^ALDH^hi^Lin^-^) by a single intravenous (IV) injection via the tail vein. PBS injected or control animals (no AMI) were analyzed in parallel. Mice were sacrificed 48 - 72 hours post transplantation and organs were harvested, rinsed in PBS and analyzed on a Kodak 4000 MM CCD/X-ray imaging station (Molecular Imaging Systems, Eastman Kodak Company, New Haven, CT) as described[17]. Relative intensities were measured by comparing regions of interest (ROI) applied to the tissue images. ROI values of untreated controls were defined as 1.

### Four week transplantation experiment

NOD/SCID β2m null mice to be transplanted were subjected to AMI on the day before transplantation, as described[18]. Human UCB Lin^- ^cells were sorted according to high or low expression of ALDH as described above and 0.5-1 × 10^6 ^ALDH^lo^Lin^- ^(n = 6) or 0.6-1 × 10^6 ^ALDH^hi^Lin^- ^(n = 11) cells or PBS (n = 13) was transplanted by a single IV injection. Mice were sacrificed 28 days post transplantation and organs were harvested and processed for frozen sectioning.

### Echocardiography

Transthoracic echocardiography was performed in anesthetized mice by using an Acuson Sequoia 256 Echocardiography System (Acuson Corp., Mountain View, California, USA) equipped with a 15-MHz (15L8) transducer as previously described [19]. Ejection fraction (EF), left ventricular end diastolic volume (LV-EDV), left ventricular end systolic volume (LV-ESV), and segmental wall motion scoring index (SWMSI) were evaluated on the day of transplantation (day 1 post surgery) and at one and four weeks post transplantation as described[20]. Animals were stratified into groups with small, medium and large infarcts, as described [20]. The echocardiographer was always blinded to the specific treatments of the animals.

### Immunofluorescence

Hearts, spleens, lungs, livers, and kidneys were quickly removed and placed in PBS at room temperature for 5 minutes to allow excess blood to drain out. The organs were then placed in ice-cold PBS and processed for frozen sectioning. Hearts were cut into three transverse sections in a bread loaf manner and embedded in O.C.T compound before rapid freezing in liquid nitrogen cooled acetone/methanol. Spleens and sections from livers, lungs, and kidneys were processed in parallel. 5 μm frozen sections were mounted on Superfrost microscope slides. Human cells were detected using human specific antibodies: rabbit anti-β2-Microglobulin (1:800, Abcam, Cambridge, United Kingdom), mouse anti-CD45 (1:200, Vector Laboratories, Burlingame, CA) and mouse anti-CD31 (1:100, DAKO, Glostrup, Denmark). Staining was visualized using highly cross-adsorbed goat anti-mouse or anti-rabbit secondary antibodies conjugated with either Alexa488 or Alexa594 antibodies (1:000, all Invitrogen) and sections were mounted with DAPI containing Neomount mounting medium (Invitrogen). Relevant isotype controls were stained in parallel. Comparable frozen sections of hearts from PBS injected mice or human heart were used as negative and positive controls, respectively. Sections were analyzed on a Zeiss Axiovert4000 wide field fluorescent microscope (Carl Zeiss Inc., Oberkochen, Germany) using the Metamorph software (Molecular Devices, Sunnyvale, CA). Image stacks of thin serial sections were obtained from selected sections by Z-stage scanning. Blinded 3D deconvolution (Autoquant, Media Cybernetics, Inc., MD) was used to reduce out of focus light and enhance signal to noise ratio. Single thin optical sections were generated using the ImageJ software (Rasband, W.S., ImageJ, U. S. National Institutes of Health, Bethesda, Maryland, USA, http://rsb.info.nih.gov/ij/, 1997-2006).

### Vascular density

5 μm frozen sections from the basal and medial portion of the hearts from each treatment group (PBS: n = 12; ALDH^lo^Lin^-^: n = 5; ALDH^hi^Lin^-^: n = 9) were stained with mouse-specific rat anti-CD31 antibody (1:100, BD Biosciences, San Diego, CA) and visualized using a HRP-conjugated secondary goat anti-mouse antibody (Acriz Antibodies GmbH, Hiddenhausen, Germany) and DAB+ chromagen according to the manufacturer's instruction (DAKO). For each heart, bright field images were recorded from 10 randomly selected visual fields (40× magnification) in the tissue sub-served by the infarct related artery. Mean vascular density per μm^2 ^tissue was estimated for each group. Only CD31 positive structures with a well defined tubular morphology or structures with a linear extension equal to or larger than 50 μm were scored as positive. Images were analyzed using the ImageJ software.

### Statistical analyses

All data were analyzed by ANOVA with Bonferroni correction for multiple comparisons. p-values smaller than or equal to 0.05 were considered significant. Hadis method to identify outliers in multivariate data[21] was applied to the vascular density data with a 95% significance level.

## Results

### Distribution of ALDH^lo^Lin^-^, ALDH^hi^Lin^-^, and CD34^+ ^cells at 48-72 hours post transplantation

**Figure 1:**
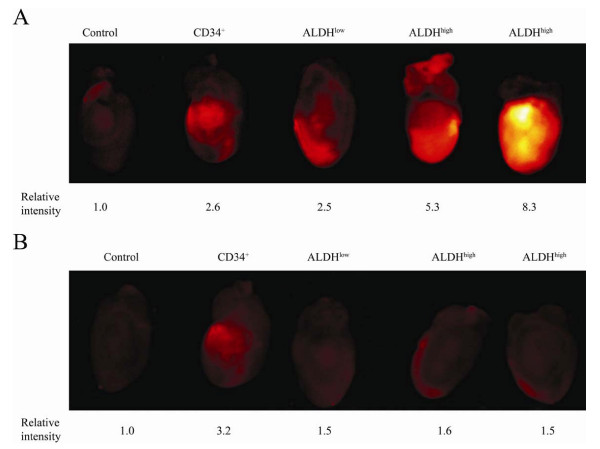
**Distribution of human UCB CD34^+^, ALDH^lo^Lin^-^, or ALDH^hi^Lin^- ^sorted cells to the site of injury in NOD/SCID mice with AMI**. AMI was induced in NOD/SCID mice by permanent ligation of the LAD. On the following day, animals were transplanted with 2 × 10^6 ^CD34^+^, 4 × 10^5 ^ALDH^lo^Lin^-^, or 4 × 10^5 ^ALDH^hi^Lin^- ^UCB cells labeled with QD655 fluorescent nanoparticles. Hearts were removed 48 hours post transplant and near infra-red images were recorded. (**A**) Anterior wall, (**B**) posterior wall. Values indicate relative fluorescent intensity. Value of the control is set at 1.

We first evaluated the short term homing potential of three human stem and progenitor cell populations, ALDH^hi^Lin^-^, ALDH^lo^Lin^-^, and CD34^+^, purified from UCB as previously described[8]. Purified cells were labeled with QD655 or Feridex750 fluorescent particles (2 × 10^6 ^CD34^+^, 1.6 - 4 × 10^5 ^ALDH^lo^Lin^-^; 2.3 - 4 × 10^5 ^ALDH^hi^Lin^-^), transplanted to NOD/SCID or NOD/SCID β2m null mice with surgically induced AMI and selected organs were analyzed on a Kodak 4000 MM CCD/X-ray imaging station 48-72 hours post transplantation as described[17] (Figure 1). We found greater signal intensity at the site of injury in the hearts of ALDH^hi^Lin^- ^cell treated animals, as compared to ALDH^lo^Lin^- ^cell treated mice (Figure 1A). Donor cells were predominantly located at the site of injury as evident from images taken of the posterior, non-infarcted wall (Figure 1B). Although based on limited data, it was also interesting to note that CD34^+ ^cells, although representing a major sub-population in the ALDH^hi^Lin^- ^fraction, did not appear to home with the same specificity or robustness. To exclude the possibility that the fluorescent signal was derived from contaminating free nanoparticles co-injected with the donor cells, we sorted for high or low ALDH expression after labeling with Feridex750 nanoparticles and prior to transplantation. As can be seen in Additional file 1, we confirmed the preferential infarct specific distribution of the ALDH^hi^Lin^- ^sorted cells. Interestingly, using cells purified after Feridex nanoparticle labeling, it could be observed that ALDH^lo^Lin^- ^cells, which represent a committed progenitor population, appeared to traffic to the spleen at greater frequency in comparison to ALDH^hi^Lin^- ^cells, as evident from the higher fluorescent intensity in the spleens of animals transplanted with ALDH^lo^Lin^- ^cells, as compared to animals that received ALDH^hi^Lin^- ^cells. In contrast, as also seen in figure 1, the more primitive ALDH^hi^Lin^- ^stem cell population preferentially homed to the infarcted heart.

### Multi-organ engraftment

Next, we evaluated the engraftment and regenerative potential of highly purified ALDH^lo^Lin^- ^and ALDH^hi^Lin^- ^cells that had been FACS sorted from human Lin^- ^UCB in NOD/SCID β2m null mice with surgically induced AMI four weeks post transplant (ALDH^lo^Lin^- ^(n = 6) or ALDH^hi^Lin^- ^(n = 11) cells or PBS (n = 13)).

**Figure 2:**
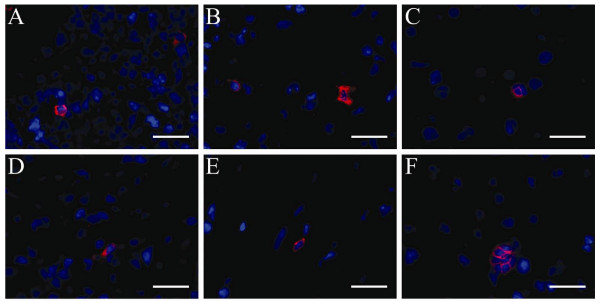
**Multi-organ engraftment in NOD/SCID β2m null mice four weeks after transplantation of ALDH^hi^Lin^- ^sorted human UCB cells**. NOD/SCID β2m null mice with AMI were transplanted with ALDH^hi^Lin^- ^sorted human UCB cells and human engraftment in multiple organs was assessed by staining for human specific β2m four weeks post transplant. (**A**) Spleen, (**B**) lung, (**C**) liver, (**D**) kidney, (**E**) heart, (**F**) liver. Nuclei: blue, β2m: red. Scale bar represents 25 μm.

The NOD/SCID β2m null mouse strain is null for the MHC-I associated β-2-microglobulin gene product that is expressed on all nucleated cells. This allowed us to specifically detect human cells regardless of phenotypic fate in the murine background by antibody-mediated staining for β2m. Sections from spleen, lung, kidney, liver and heart revealed human engraftment in 10 of 11 ALDH^hi^Lin^-^transplanted animals (Figure 2) and in four of six ALDH^lo^Lin^- ^transplanted animals (data not shown). The human engraftment in the ALDH^hi^Lin^-^transplanted animals was generally more widespread with human cell present in the spleen, lung, liver, heart, and kidney. Only sporadic human cells were detected in ALDH^lo^Lin^- ^transplanted animals and never in multiple organs of the same animal (data not shown). Engrafting human cells appeared small and round to oval shaped with a small cytoplasm relative to the nucleus. Engraftment appeared evenly dispersed throughout the tissues, mostly as single cells and only rarely in clusters of two or more cells (Figure 2F).

**Figure 3:**
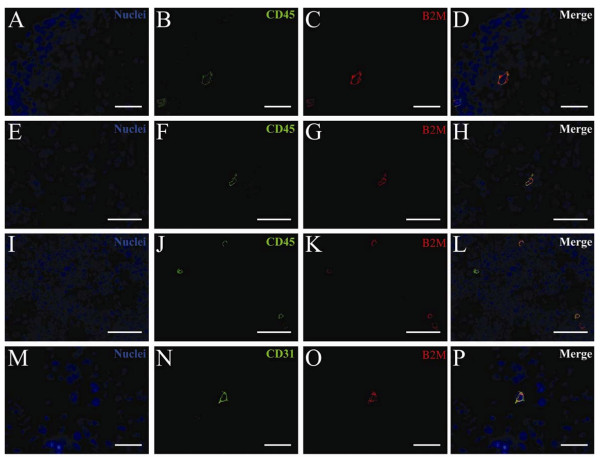
**Multi-lineage human engraftment in selected organs in NOD/SCID β2m null mice four weeks after transplantation of ALDH^hi ^Lin^- ^sorted human UCB cells**. NOD/SCID β2m null mice with AMI were transplanted with ALDH^hi^Lin^- ^sorted human UCB cells. The lineage of human engrafting cells in selected organs was assessed by double staining for human-specific β2m and CD45 (**A-L**) or CD31 (**M-P**) four weeks post transplantation. (**A-D**) Lung, (**E-H**) Kidney, (**I-L**) Spleen, (**M-P**) Lung. Nuclei: blue, CD45 and CD31: green, β2m: red. Scale bar represents 25 μm.

Engrafting human cells were further characterized by double staining for human-specific β2m in combination with either a human-specific CD45 pan-leukocyte antibody or a human-specific CD31 endothelial antibody. CD45 positive cells accounted for the majority of the engrafting cells (Figures 3A-L). We found very few donor derived CD31 positive cells (representative staining from the lung shown in Figures 3M-P).

### Cardiac engraftment

**Figure 4:**
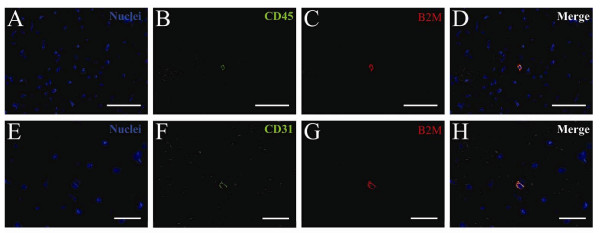
**Human engraftment in the heart of NOD/SCID β2m null mice with AMI four weeks after transplantation of ALDH^hi ^Lin^- ^sorted human UCB cells**. NOD/SCID β2m null mice with AMI were transplanted with ALDH^hi^Lin^- ^sorted human UCB cells. The lineage of human engrafting cells in selected organs was assessed by double staining for human specific β2m and CD45 (**A-D**) or CD31 (**E-H**) four weeks post transplantation. Nuclei: blue, CD45 and CD31: green, β2m: red. Scale bar represents 25 μm.

We analyzed hearts from the two cell-treated groups in greater detail. To estimate the level of engraftment, we identified β2m-positive nucleated human cells in a total of 150 individual sections obtained from the basal, medial, and apical portions of the hearts. Human engraftment in the heart, defined as the presence of at least three individual β2m- positive cells in the combined tissue analyzed from the basal, medial, or apical sections, was seen in 10 of 11 ALDH^hi^Lin^- ^transplanted animals. Human cardiac engraftment was determined by PCR on purified DNA from thin frozen sections as described[22] and revealed that all of the ALDH^hi^Lin^- ^treated animals but none of the ALDH^lo^Lin^- ^treated animals were positive for human specific Alu sequence. We have recently reported this same phenomenon in the liver, with only the ALDH^hi ^cells homing to the site of tissue damage, as verified by FACS and ALU analysis[23]. Human cells were found in only one of the ALDH^lo^Lin^- ^transplanted animals. For each section analyzed, we found 1 to 10 human cells in the hearts of ALDH^hi^Lin^- ^cell-transplanted animals. The human cells were primarily found as individual cells located in the non-infarcted healthy myocardium (Figure 4) and only rarely in the infarcted tissue or infarct border. Occasionally two or three cells were found clustered together. The human cells were small and round to oval shaped with a small cytoplasm relative to the nucleus. We found no cells with cardiomyocyte morphology in the 150 individual sections analyzed. Staining for human hematopoietic and endothelial cells with human-specific CD45 or CD31 antibodies, respectively, revealed a pattern similar to that found in the lung, liver, kidney, and spleen. The majority of the human cells co-expressed CD45 (Figure 4D) while β2m/CD31 double positive human cells were rare and not integrated in the epithelium of large caliber vessels (Figure 4H).

### Functional recovery

We have previously shown that the initial infarct size in the murine AMI model is critical for the disease progression and late infarct size[20]. Thus, animals that only receive a small infarct recover easily from injury to levels comparable to sham operated controls. Stratifying the mice based on the day 0 infarct size in the present study did not, however, influence the interpretation of the data and all transplanted animals were included in the final evaluation.

**Figure 5:**
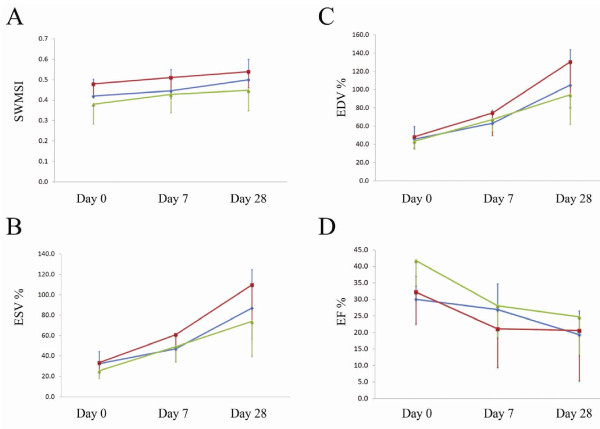
**Cardiac function of NOD/SCID β2m null mice with AMI four weeks after transplantation of ALDH^lo ^Lin^- ^or ALDH^hi ^Lin^- ^sorted human UCB cells or PBS**. NOD/SCID β2m null mice with AMI were transplanted with ALDH^lo^Lin^- ^(Red square) or ALDH^hi^Lin^- ^(Green triangle) sorted human UCB cells or PBS (Blue diamond). Echocardiographic images were recorded on the day of transplantation (day 0) and again at day 7 and day 28. Segmental wall motion scoring index (A), end diastolic volume (B), end systolic volume (C), and ejection fraction (D) were determined. Data points indicate mean values and standard error.

NOD/SCID β2m null mice with AMI were transplanted with ALDH^lo^Lin^- ^(Figure 5 - Red square) or ALDH^hi^Lin^- ^(Figure 5 - Green triangle) sorted human UCB cells or PBS (Figure 5 - Blue diamond). Serial echocardiographic images were recorded for all treatment groups (PBS, ALDH^lo^Lin^-^, and ALDH^hi^Lin^-^) on the day following surgery (day 0) and again at one and four weeks post transplantation. All treatment groups had similar sized infarcts at the time of transplantation, as evident from day 0 SWMSI. There was no improved cardiac function at the experimental end point. At four weeks, we thus found no significant difference in EF, LV-EDV, LV-ESV or SWMSI between any of the treatment groups (Figure 5).

### Vascular density

**Figure 6:**
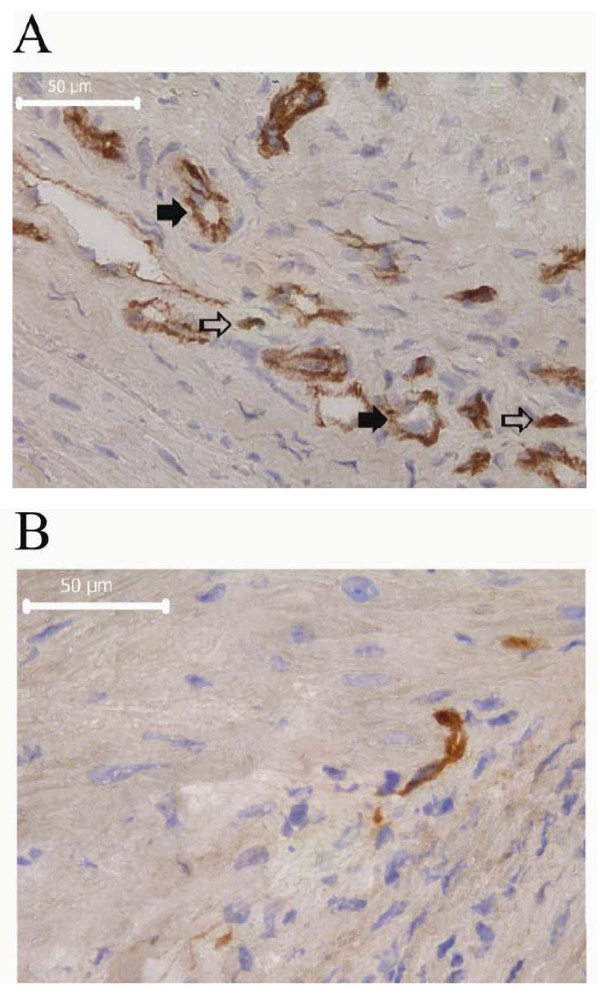
**Vascular density in the infarct zone of NOD/SCID β2m null mice with AMI four weeks after transplantation of ALDH^lo ^Lin^- ^or ALDH^hi ^Lin^- ^sorted human UCB cells**. NOD/SCID β2m null mice with AMI were transplanted with ALDH^lo^Lin^- ^or ALDH^hi^Lin^- ^sorted human UCB cells or PBS. Frozen sections were stained with a mouse specific CD31 antibody and visualized with DAB+ chromagen. Ten high power fields were recorded from each heart (PBS: n = 12; ALDH^lo^Lin^-^: n = 5; ALDH^hi^Lin^-^: n = 9) in the tissue sub served by the infarct related injury. Representative CD31 labeling from the infarct zone of an ALDH^hi^Lin^- ^or ALDH^lo^Lin^- ^transplanted animal are shown in (A) and (B), respectively. Arrows point to representative CD31 stained structures that were excluded (open arrows) or included (solid arrows) in the estimation of vascular density. See text for further explanation. Nuclei: blue, CD31: brown. Scale bar represents 50 μm.

We analyzed whether the transplanted cells promoted re-vascularization of the infarcted tissue by host endothelial cells. Sections were stained with a murine-specific CD31 endothelial antibody and we evaluated the mean vascular density in the infarcted tissue sub-served by the infarct related artery normalized to the μm^2 ^tissue analyzed. CD31 is expressed on platelets and a number of hematopoietic cell types that infiltrate infarcted tissue including macrophages, neutrophils, and NK cells[24]. To avoid the potential inclusion of non-endothelial cell types (Figure 6, open arrows) in the estimation of vascular density, we only counted CD31 positive structures with a well defined tubular morphology or an open lumen, or structures with a linear extension equal to or larger than 50 μm (Figure 6, solid arrows). We found a mean capillary density of 6.0, 5.4, and 4.1 large caliber vessels pr. 1000 μm^2 ^tissue in the ALDH^hi^Lin^-^, ALDH^lo^Lin^- ^and PBS treated groups, respectively (95% confidence interval [5.0-7.0], [4.4-6.5], [3.3-5.0]; Table 1). We found a significant increase in capillary density in the ALDH^hi^Lin^- ^treated group as compared to the PBS treated group at four weeks post transplantation (p = 0.011 versus PBS; Table 1). Although the ALDH^lo^Lin^- ^treated group was not significantly different from the PBS treated group, we noted a tendency toward an intermediate improvement in vascular density in the ALDH^lo^Lin^- ^treated groups. Using the Hadis method to identify outliers in multivariate data[21] with a 95% significance level eliminated two high power fields in the PBS treated groups and one outlier in the ALDH^hi^Lin^- ^treated group. Between group comparison after elimination of outliers revealed that both the ALDH^hi^Lin^- ^treated and the ALDH^lo^Lin^- ^treated groups were significantly different from the PBS treated group (p = 0.001 and p = 0.031, respectively).

**Table 1:** Mean vascular density in the infarct zone of NOD/SCID β2m null mice with AMI four weeks post transplant of PBS, ALDH^lo ^Lin^- ^or ALDH^hi ^Lin^- ^sorted human UCB cells

Treatment^a^	n^b^	Mean vascular density/1000 μm^c^	95% Confidence interval	p versus PBS
PBS	12	4.1	[3.3-5.0]	-
ALDH^lo^Lin^-^	5	5.4	[4.4-6.5]	0.279 (0.031)^d^
ALDH^hi^Lin^-^	9	6.0	[5.0-7.0]	0.011 (0.001)^d^

^a ^NOD/SCID β2m null mice with AMI were transplanted with ALDH^lo^Lin^- ^or ALDH^hi^Lin^- ^sorted human UCB cells or PBS. Frozen sections were stained with a mouse specific CD31 antibody and visualized with DAB+ chromagen.

^b ^Number of hearts analyzed pr. group; 10 randomly selected visual fields (40× magnification) in the tissue sub-served by the infarct related artery were analyzed from each heart.

^c ^CD31 positive vascular structures with a well defined tubular morphology or an open lumen or structures with a linear extension equal to or larger than 50 μm were included.

^d ^p-value after correction for outliers.

## Discussion

In the current studies we have adapted the LAD occlusion model of AMI to immune deficient NOD/SCID and NOD/SCID β2m null mice. We used this model to evaluate the global engraftment potential of purified human UCB cell populations as well as the distribution, engraftment, and regenerative potential for the infarcted heart.

We first used fluorescent nanoparticle labeling to trace the donor cell distribution to various organs, including the infarcted myocardium, following IV injection. We have recently documented that sorting of the labeled cells is essential to avoid infusing large numbers of unbound nanoparticles[17]. Non-cell mediated splenic sequestering of fluorescent nanoparticles was indeed pronounced in our previous report when control NOD/SCID β2m null mice received free 750 nm fluorescently conjugated Feridex nanoparticles[17]. The fluorescent intensities found in the NOD/SCID mice transplanted with QD655 labeled cells in the present study may thus include both cell specific and unspecific non-cell mediated fluorescence. Our present results from animals transplanted with 750 nm Feridex labeled cells sorted prior to infusion, however, confirm a significant distribution of labeled donor cells to the infarcted tissue in the absence of nonspecific signal from free nanoparticles. We have previously found a labeling efficiency between 28% and 40% with fluorescently conjugated Feridex nanoparticles, depending of the purification method[17]. Specifically, the Feridex labeling efficiency of UCB CD34^+ ^purified cells was approximately 32% while Lin^- ^purified UCB cell labeled at approximately 39%. Although we did not measured the QD655 and Feridex nanoparticle labeling efficiency of the Lin^-^ALDH^hi ^and Lin^-^ALDH^lo ^purified cells used in the present study, we expect that differential labeling efficiency is not responsible for the observed difference in signal intensity. Although we were clearly able to visualize a specific trafficking of ALDH^hi^Lin^- ^cell to the site of injury, we were unable to image the organs non-invasively thus precluding a longitudinal evaluation of donor cell distribution. Using a similar cell sorting and labeling strategy we, however, recently demonstrated that donor cells could be detected in the ischemic hind limb up to seven days after transplantation[17]. The difference in sensitivity between our previous study and the present one is likely due to interference from the additional overlying tissue of the thoracic cavity and localized transplantation and/or labeling with fluorescent nanoparticles emitting in the far red range may be needed in order to improve tissue penetration and allow non-invasive visualization of labeled cells in situ[17]. Also, the electron-dense properties of the fluorescent nanoparticles presently employed potentially allow for multimodal non-invasive visualization of labeled cells using both fluorescent and magnetic resonance imaging[17]. We have also recently worked with perfluorocarbon nanobeacons, which have a higher emission and penetrance without background and might be better suited for in vivo imaging of deep tissues[17].

Both the NOD/SCID and the NOD/SCID β2m null strains presently used are known to support multi-lineage engraftment of human hematopoietic cells. Identification of engrafting human cells in solid organs is, however, difficult and requires labeling of donor cells prior to transplantation by ex vivo manipulation of target cells prior to transplantation or by application of complex immunoassay techniques. Extensive ex vivo manipulation of the donor cells is undesirable and may adversely affect the cells and increase the risk of contamination while antibody staining for specific human lineage markers typically requires knowledge of the expected differentiation pattern of the transplanted cells, so unexpected cell phenotypes may go unnoticed. Antibody staining for β2m is, on the other hand, quick and versatile, and requires no ex vivo manipulation of the donor cell. Moreover, no nonspecific staining of endogenous β2m is seen in NOD/SCID β2m null strain and donor derived cells are detected regardless of post transplantation phenotypic fate. A drawback of the β2m staining approach relates to the possible down regulation of β2m expression by some types of cancer cells as a mechanism to avoid normal host cancer surveillance[25]. Although we are not aware of any literature describing a similar down regulation of β2m expression by non-carcinogenic cells in the setting of xenogeneic transplantation, we cannot exclude the fact that we may underestimate the number of engrafting human cells by this method. To compensate for this shortcoming and to confirm the human specificity of our β2m staining, we employed human specific lineage specific antibodies throughout the study. Alternatively, we have also recently described an alternative murine xenograft model based on the β-glucuronidase (GUSB) deficient NOD/SCID/MPSVII mouse strain[17,23]. The lack of GUSB expression by the host tissue similarly allows rapid and precise identification of engrafting human cells by staining for donor GUSB activity. Using the NOD/SCID/MPSVII model, we demonstrated multi-organ engraftment of human UCB-derived ALDH^hi^Lin^- ^cells 10-12 weeks post transplantation[11]. Both the present model and the NOD/SCID/MPSVII model are thus ideally suited for pre-clinical evaluation of prospective cell populations and application strategies in cell-based regenerative therapy.

We and others have previously shown that ALDH^hi^Lin^- ^cells have a superior hematopoietic repopulating potential in the BM and spleen of NOD/SCID and NOD/SCID β2m null mice, as compared to CD34^+ ^or ALDH^lo^Lin^- ^cells [7-10]. ALDH^lo^Lin^- ^cells are, as verified in the present study, indeed virtually devoid of long term repopulation potential. In addition, we have recently shown that ALDH^hi^Lin^- ^sorted cells from human BM contained populations of functionally primitive mesenchymal progenitor populations[26]. UCB, as used in the present study, is, however, known to contain lower numbers of mesenchymal progenitors in comparison to BM[17]. We cultured the cells overnight under conditions that promote retention of primitive hematopoietic phenotypes[17]. The present AMI xenotransplantation study thus predominantly reflects the regenerative potential of highly purified hematopoietic stem and progenitor cells. Gentry et al. have previously shown that ALDH^hi ^sorted cells contain subsets of primitive stem and progenitor cells of non-hematopoietic lineages, including mesenchymal stem cells and endothelial progenitor cells[6]. Although we did not assess the proportion of these non-hematopoietic cells in the present study, due to the cell source and isolation and culture method, it is unlikely that they contributed to the observed results in a substantial way. We found no evidence of a direct contribution of the transplanted cells to regenerated infarcted tissue although down regulation of β2m expression by the donor cells as discussed above may have rendered some donor-derived cells types undetectable by our present methods. Engrafting human cells were predominantly of a hematopoietic phenotype, although non-hematopoietic cells were also identified. These CD45 negative cells rarely appeared in the infarcted tissue and it is therefore unlikely that they represent primitive cardiomyocytes. We were unable to precisely determine if the engrafting cells were tissue resident cells or circulating hematopoietic cells retained in the microvasculature. Although none of the donor cells appeared to reside in large caliber vessels we did, however not analyze peripheral blood samples to confirm the presence of a circulating pool of donor derived cells. Moreover, although we recently reported that fusion of human donor UCB ALDH^hi^Lin^- ^cells and host murine hepatocytes could generate hybrid cells that only retained minimal amounts of human DNA in a NOD/SCID/MPSVII liver injury model, this was indeed a very rare event[23]. The present results are thus more in line with our previous results and recent reports on the role of donor hematopoietic cells in the regeneration of damaged tissue[17,26-28]. In a recent study we also failed to detect any long term human myocardial engraftment or functional improvement following intramyocardial injection of human CD34^+ ^sorted mobilized peripheral blood progenitors in athymic nude rats with AMI[29]. In the present study we were similarly unable to detect an improvement in cardiac function as a result of cell treatment in either the ALDH^lo^Lin^- ^or ALDH^hi^Lin^- ^treated groups. We did, however detect a significantly better vascularization of the central infarct area in the ALDH^hi^Lin^- ^treated group as compared to the ALDH^lo^Lin^- ^and PBS treated groups. The fact that the ALDH^lo^Lin^- ^cells also appeared to improve vascular density compared to PBS when correcting for outliers suggested that this population, although devoid of long term repopulating cells, may include a transiently present population of cells with angiogenic potential.

The most well described larger randomized clinical study of cell-based regenerative therapy for AMI reports a modest 2.5% increase in left ventricular EF following intra-coronary infusion of BM MNCs[30]. We were unable to detect an improvement in cardiac function as a result of cell treatment in either the ALDH^lo^Lin^- ^or ALDH^hi^Lin^- ^treated groups. It should, however, be noted that the study was not powered to detect small improvements in cardiac function and modest improvements as reported in clinical trials would thus go unnoticed in the present study. The fact that we found a superior vascularization in the ALDH^hi^Lin^- ^treated group but no improvement in cardiac function may indeed be due to the relatively large variation in the echocardiographic data. The lack of a detectable functional improvement can, alternatively, be explained by the early end point of functional evaluation. It is indeed at this point not clear whether the vascular structures that we detected in the central infarct area are patent and thus represent mature and functional blood vessels. These questions may be resolved in future studies by both including a more direct measure of blood flow to the infracted area as well as extending the evaluation period to eight weeks and beyond. Nonetheless, a long term benefit is not likely to depend on a direct contribution of the transplanted cells to the regenerating myocardium, since we found no evidence of a substantial donor derived population in the central infarct area or in the blood vessels. These results are in agreement with our recent findings that human BM derived ALDH^hi^Lin^- ^cells improve perfusion to the ischemic hind limb of NOD/SCID β2m null mice and improve vascular density as compared to ALDH^lo^Lin^- ^or MNC control treated mice[26]. Moreover, using a similar labeling strategy as the one employed in the present study, we found that the human donor cells only transiently engrafted the ischemic tissue. Only few cells were detected at 21 to 28 days post transplant in animals receiving ALDH^hi^Lin^- ^cells while animals receiving ALDH^lo^Lin^- ^cells were devoid of engrafting donor cells at the endpoint. Although there are obvious differences with respect to the cell source and the details of the purification protocols employed in our hind limb ischemia study and the present study, the lack of long term engraftment of the ALDH^lo^Lin^- ^cells as shown in the hind limb ischemia model is corroborated by the present immunofluorescent and PCR data. The sensitivity of our PCR assay may however allow for a non-detected low level of engraftment to persist although we have previously been able to detect ~2 human cells per 10.000 murine cells in a related PCR system[31]. Even though we found similar results using UCB and BM in the present cardiac infarction models and in our previously reported hind limb ischemia model, respectively, in a direct comparison of BM and UCB derived human CD133^+ ^purified cells, Ma *et al *found that only BM derived cells induced functional recovery as measured by improved shortening fraction at four weeks post intramyocardial transplantation of 5 × 10^5 ^human donor cells in a NOD/SCID cryo-injury model of AMI[32]. Interestingly, in spite of the significant difference in functional recovery between UCB and BM treated animals, no difference was observed in infarct size and capillary density between the two cell treatment groups.

In conclusion, we found that a larger proportion of human UCB cells selected according to high expression of the cytosolic enzyme aldehyde dehydrogenase specifically distributed to the infarcted tissue as compared to cells with low aldehyde dehydrogenase activity. ALDH^hi^Lin^- ^cells also had a superior global engraftment potential in multiple organs including the infarcted heart at four weeks post transplantation. Although no significant improvement in cardiac performance was detected at four weeks post transplantation, the superior engraftment potential was associated with an increased vessel density in the infarct zone, as compared to controls. The significant increase in vessel density in the stem cell-injected mice, as compared to the injured but non-transplanted, or committed progenitor - transplanted controls, is interesting, and the mechanism responsible is not yet known. The increased density of large-caliber vessels could be caused by an enlargement in size and function of pre-existing tiny vessels, or could be caused by neovascularization into the infarct zone. Future studies will examine those possibilities.

## Competing interests

The authors declare that they have no competing interests.

## Authors' contributions

CSS and DH conceived of the study and carried out its design and coordination. DM and DPW were responsible for imaging studies. CW performed LAD ligation to promote cardiac injury. IR assisted in stem cell isolation and Flow cytometry. MC provided umbilical cord blood samples discarded form the St. Louis cord blood bank and reviewed data. AK performed functional cardiology studies in the murine recipients of the human stem cells. LP and JAN funded the study, approved of its design, reviewed and interpreted the data. CSS and JAN wrote the manuscript and performed editorial revisions. All authors read and approved the manuscript.

## Supplementary Material

Additional file 1 **Distribution of human UCB ALDH^lo^Lin^-^, or ALDH^hi^Lin^- ^nanoparticle-labeled and re-sorted cells to the site of cardiac injury vs. spleen in NOD/SCID β2m null mice with AMI**. AMI was induced in NOD/SCID β2m null mice by permanent ligation of the LAD. On the following day, animals were transplanted with 2 × 10^6 ^CD34^+^, 4 × 10^5 ^ALDH^lo^Lin^-^, or 4 × 10^5 ^ALDH^hi^Lin^- ^UCB cells that had been labeled with Feridex750 fluorescent nanoparticles and then sorted to remove unbound particles. Hearts were removed 48 hours post transplant and near infra-red images were recorded. (**A**) Anterior wall-infarct site, (**B**) spleen lodgment. Values indicate relative fluorescent intensity. Value of the control is set at 1. Click here for file
