# Supplementary material for: Human cord blood progenitors with high aldehyde dehydrogenase activity improve vascular density in a model of acute myocardial infarction
Source: J Transl Med. 2010 Mar 9;8:24. doi: 10.1186/1479-5876-8-24 (PMC2846892; doi:10.1186/1479-5876-8-24)
Supplement: Additional file 1 — Distribution of human UCB ALDHloLin-, or ALDHhiLin- nanoparticle-labeled and re-sorted cells to the site of cardiac injury vs. spleen in NOD/SCID β2m null mice with AMI. AMI was induced in NOD/SCID β2m null mice by permanent ligation of the LAD. On the following day, animals were transplanted with 2 × 10^6^ CD34+, 4 × 10^5^ ALDHloLin-, or 4 × 10^5^ ALDHhiLin- UCB cells that had been labeled with Feridex750 fluorescent nanoparticles and then sorted to remove unbound particles. Hearts were removed 48 hours post transplant and near infra-red images were recorded. (A) Anterior wall-infarct site, (B) spleen lodgment. Values indicate relative fluorescent intensity. Value of the control is set at 1. [file 1479-5876-8-24-S1.PDF]

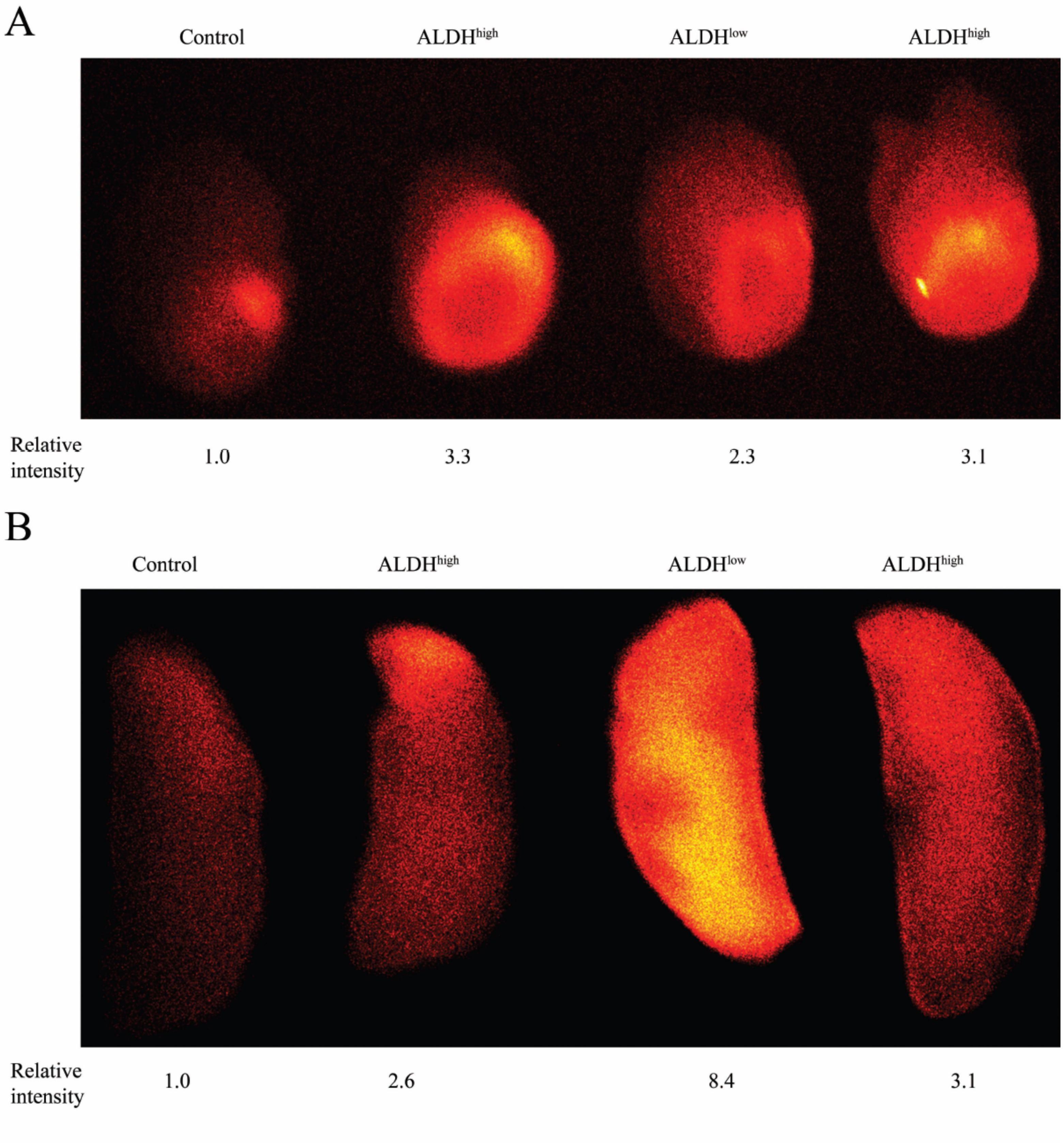

A
Control
ALDHhigh
ALDHlow
ALDHhigh
Relative intensity
1.0
3.3
2.3
3.1
B
Control
ALDHhigh
ALDHlow
ALDHhigh
Relative intensity
1.0
2.6
8.4
3.1
